# Efficiency in the evolution of metro networks

**DOI:** 10.1038/s41598-022-12053-3

**Published:** 2022-05-18

**Authors:** Aihui Pei, Feng Xiao, Senbin Yu, Lili Li

**Affiliations:** 1 Research Center of Logistics, Research Institute of Highway, Ministry of Transport, Beijing, 100088 China; 2 College of Engineering, Key Laboratory of Urban Rail Transit Intelligent Operation and Maintenance Technology & Equipment of Zhejiang Province, Zhejiang Normal University, Jinhua, 321004 China; 3 College of Engineering, Zhejiang Normal University, Zhejiang, 321004 China; 4 Chengdu Metro Operation Co, Ltd, Chengdu, 610031 China

**Keywords:** Complex networks, Statistical physics

## Abstract

Metro systems extended rapidly in China, especially in the last decade, developing over a half-century. This work explores the dynamical evolution of the structural efficiency of metro systems interpreted as complex networks for 14 large cities in mainland China. Based on the empirical observations, we find that the global efficiencies scale with the number of stations and counter-intuitively decreases as the metro networks expand, which shows a long-tail characteristic. The evolution of metro networks is, in essence, the improvement of the relative ratio of average nodal efficiency in the core compared to global efficiency. These relationships are in good agreement with the temporal structure of metro networks. Besides, we find that the metro stations with the higher efficiencies are those surrounding the urban center, and most of them dwell within the core and gradually expand the branches in space. Our findings suggest that the evolution properties of metro systems influenced by numerous geographical, historical, and social activities suggest that underlying, universal mechanisms are at play during their evolution in the spatial–temporal dimension.

## Introduction

Metro systems are integral parts of transit systems in large cities, acting as a critical solution in supporting commuter traffic demand within the metropolis area^1^. Compared with other transportation modes, metro systems offer an effective solution for addressing the urban issues of being safer, punctual, quicker, cleaner, land-saving, and high capacity. The dependence on metro systems keeps growing in cities worldwide as societies become ever more urbanized, which leads to a rapid increase in metro scale. This massive growth is credited to development in a few countries, especially China^2^.

As continually adding new lines and stations, the metro system, developed to improve mobility and reduce congestion in urban areas, has become an increasingly large and complex network with the city's development^3^. As one type of transportation mode, the evolution of metro networks reflects the evolution of the population and activity densities. Indeed top-down planning controlled by a central authority plays a vital role in the construction of metro networks which are always intentionally structured in a core-periphery shape. This would be true if these metro networks were planned from the beginning to their current shape, but this is not the case for most networks. The metro network results from a superimposition of multi-type actions such as urban planning, the reorganization and regeneration of economic activities, and the growth of residential populations^4^. Although central intervention is severe limitations to the possibility of modeling the dynamic metro network, top-down planning, as one of the influencing factors, could be thought of as an external perturbation^5^ when modeling a city and its expansion as a self-organized phenomenon. Understanding how metro networks evolve under different geographical, historical, and social-economic circumstances and what are the dominant mechanisms in a long time. If any, the universal features of such a large-scale self-organized process, are more critical than ever as policymakers, professionals, and the scientific communities are actively looking for new paradigms in urban planning and land management^6,7^.

The detection and characterization of these universal features require us to understand the evolution of these spatial structures. The metro network embedded in space forms a graph where stations are nodes and links represent rail connections. The evolution of the metro network is a critical aspect of the time-dependent spatial topology, which has been studied in recent years for the availability of longitudinal infrastructure data. Derrible et al.^8^ adapted graph theory to describe the metro network characteristics of the state, from, and structure. They concluded that the network topology plays a crucial role in attracting travelers to public transit. Despite their geographical and economic differences, Roth et al.^9^ found that metro networks worldwide share a common generic feature (i.e., a core with branches). However, the analysis was restricted to configurational metrics and ignored other attributes such the travel time and distances. Cats^10^ conducted longitudinal research on the topological evolution by investigating the Stockholm rail network from 1950 to 2025, which exhibited smooth long-term technological and spatial trends. Even though the growth of transportation networks is complicated and multidimensional, and the duration is usually measured in decades, it may still be tractable and predictable with a further understanding of the underlying mechanisms^11^. However, literature related to the evolution of metro networks has rarely been studied. Leng et al.^12^ proposed a new growth model composed of expanding and intensifying modes to measure the development of the transfer network of the Beijing metro. Motivated by the shape of metro networks, Aldous et al.^13^ calculated the optimal structure of metro networks as a function of the total length. They found that in the medium-length regime, resources go preferentially to radial branches as the total length increases, and this is a sharp transition when a loop appears.

The metro network plays a vital role in improving commuting efficiency and promoting economic development. The efficiency (also called *accessibility* in transport geography) measures how people interact with other people, and places were first used in transportation planning since Hansen proposed^14^. Transport operators and planners have increasingly adopted efficiency as a desirable planning goal and a key performance metric^15^. Efficiency has always been evaluated in single-mode, such as car and public transport^16,17^. A recent study proposed a new measurement approach, called Urban Accessibility Relative Index, for understanding the spatiotemporal patterns of efficiency in urban areas. Results demonstrated that the metro has a higher impact on public transit efficiency than bus^18^. The evolution of the metro network is essential to the progress of changing the opportunities of locations by metro systems, along with the developing spatial pattern, although this type of infrastructure network also lacks a high redundancy because of the high cost of adding new lines or stations. We now understand pretty well how to characterize the structure of their temporal evolution^9,19^, while transportation networks are the underlying critical infrastructures for improving the movement of people and reducing congestion in urban areas^20,21^. Unfortunately, the efficiencies changing with the structures and their evolution characteristics are generally ignored in previous studies, although the calculation and comparison of static efficiencies among metro networks appeared in several studies^22–24^.

**Figure 1:**
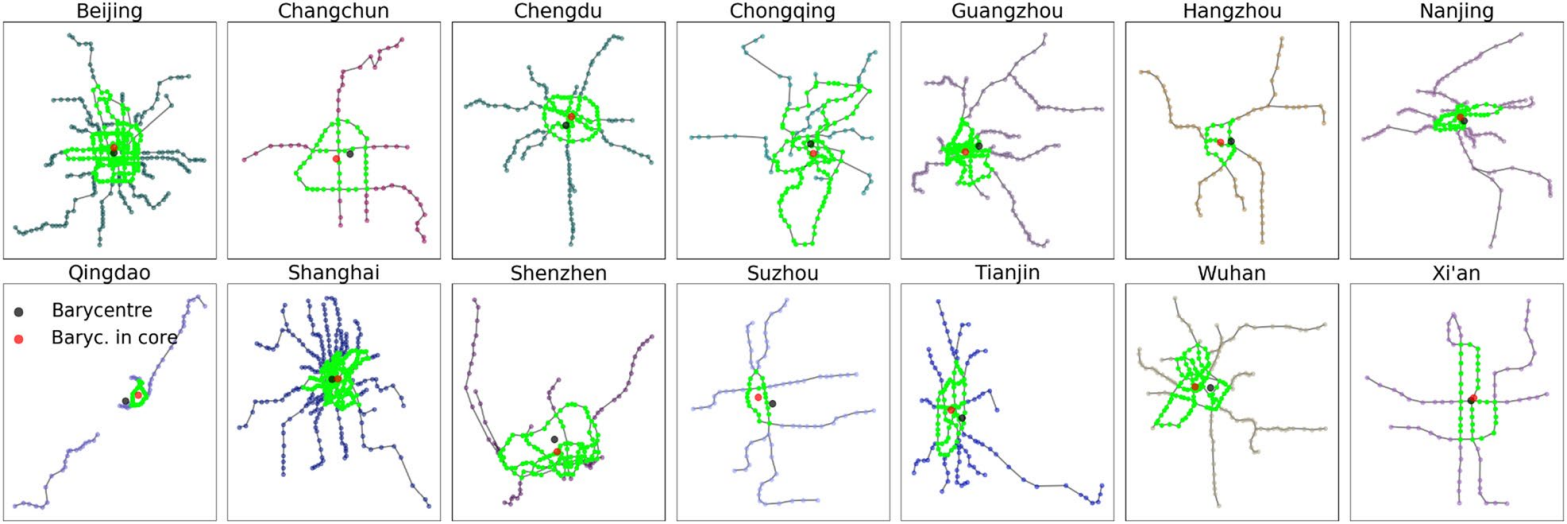
Spatial representation of 14 large metro networks in mainland China for 2019. Each city displays the core stations (marked in light green) and branch stations (marked in other colors). The red and black points denote the barycenter of core stations and the barycenter of all stations, respectively. Most core barycenter positions are located in the urban economics and politics centers, which gradually surround dense stations.

To the best of our knowledge, hardly any study has assessed network efficiency as an indicator for the evolution of empirical metro networks. This article considers the development of metro networks by investigating the structural properties and network efficiency. We focus on 14 metro networks with core (i.e., light green in Fig. 1) and branch structures identified by $k$-core ($k=2$) decomposition^9,25^ (the detail can be found in “Methods”) in mainland China. They are Beijing, Changchun, Chengdu, Chongqing, Guangzhou, Hangzhou, Nanjing, Qingdao, Shanghai, Shenzhen, Suzhou, Tianjin, Wuhan, and Xi’an, for which we offer a way to view and analyze the evolution of efficiency and related structural properties. Some early-stage metro networks in several medium-sized cities (e.g., Nanchang metro with two operating lines) are not considered here. Three sub-objectives of this study are as follows: Analyzing the evolution of topological features and global efficiency over time; Revealing the underlying cause of core-branches structure associated with urbanization and suburbanization, and the temporal evolution of efficiency in the core and on the branches; Investigating the spatial distribution and spatial organization of highest efficient nodes in metro networks.

The remainder of this paper is organized as follows: the next section presents the top results, including evolving network efficiency and the spatial organization of the highest efficient nodes. The conclusions and directions for further research are included in the last section.

## Results

### Static properties of network structure

**Figure 2:**
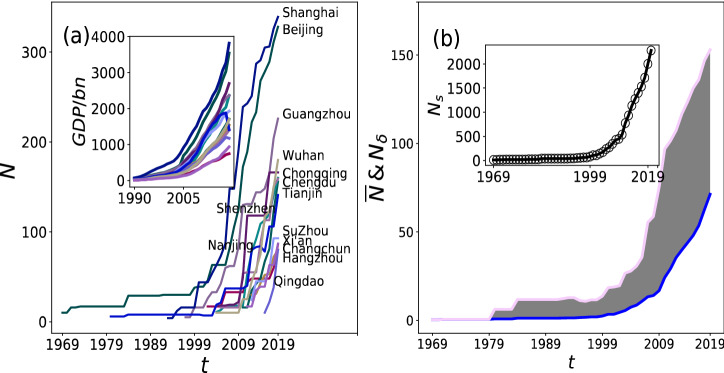
(**a**) Evolution of the network size for 14 large metro networks and GDP from 1990 corresponding to each city. (**b**) The blue curve and pink curve indicate the average number of stations $\overline{N }$ and the standard deviation (${N}_{\sigma }$) across all metro networks, respectively. The summary of stations (${N}_{s}$) in year $t$ aggregated by all networks presented in the inset.

In 1969, the oldest metro system in mainland China was built for civilian and military use in Beijing. Tianjin metro followed in 1980. Since initiating the market reform at the end of the 1970s, China has experienced rapid economic development and created a massive demand for urban transport leading to a continuing need for metro systems. Shanghai and Guangzhou began their initial operation of the metro systems in the 1990s. Then, parallel to rapid economic prosperity, as shown in the inset of Fig. 2a, Nanjing, Wuhan, Chongqing, and Shenzhen had advanced proposals waiting to be approved in the early 2000s with over hundreds of billions of GDP. Since the mid-2000s, more and more cities have drafted proposals for metro systems, rapidly appearing in 23 cities in China. The other topological characteristics and selected relevant indicators of metro networks are summarized and shown in Table 1.

**Table 1:** The summary of network indicators considered in this article is arranged according to the alphabet of city names.

City	${t}_{0}$/GDP (bn)	${M}_{l}^{{t}_{e}}$	${N}^{{t}_{e}}$	${\langle k\rangle }^{{t}_{e}}$	${\overline{l} }^{{t}_{e}}$(km)	${L}_{T}^{{t}_{e}}$(km)	${N}_{c}^{{t}_{e}}$	${\overline{r} }_{c}^{{t}_{e}}$	${\overline{r} }_{m}^{{t}_{e}}$(km)
Beijing	1969 (none)	23	328	2.26	1.67	619.10	172	12.80	25.56
Changchun	2002 (115.0)	5	86	2.07	1.08	96.34	42	7.28	13.14
Chengdu	2010 (555.1)	7	156	2.13	1.34	222.94	57	7.75	10.69
Chongqing	2004 (266.5)	10	160	2.15	1.77	305.50	113	13.94	29.20
Guangzhou	1997 (164.6)	16	226	2.15	1.93	468.72	110	10.58	23.41
Hangzhou	2012 (780.4)	4	80	2.03	1.37	112.64	22	4.30	7.41
Nanjing	2005 (241.1)	10	159	2.06	2.26	370.51	55	6.25	14.05
Qingdao	2015 (930.0)	4	80	1.98	2.15	169.57	26	6.77	13.81
Shanghai	1993 (151.9)	19	339	2.31	1.64	641.39	155	8.59	18.05
Shenzhen	2004 (342.3)	8	166	2.29	1.48	280.90	86	9.91	19.97
Suzhou	2012 (1201.2)	4	93	2.02	1.26	118.31	21	3.30	7.38
Tianjin	1980 (10.3)	6	141	2.11	1.39	209.26	61	6.19	10.13
Wuhan	2004 (195.6)	9	180	2.29	1.51	313.30	91	8.96	17.39
Xi’an	2011 (386.4)	4	87	2.05	1.40	124.46	33	4.93	8.98

${t}_{0}$ is the initial operation of the metro with the GDP of the city in that year. The other eight measures are the topological indicators for the metro network in 2019 (${t}_{e}$) including ${M}_{l}^{{t}_{e}}$ (the number of lines), ${N}^{{t}_{e}}$ (the network size), ${\langle k\rangle }^{{t}_{e}}$ (the average value of the nodal degree), ${\overline{l} }^{{t}_{e}}$ (the average interstation distance), ${L}_{T}^{{t}_{e}}$ (the total route length), ${N}_{c}^{{t}_{e}}$ (the number of nodes in the core), ${\overline{r} }_{c}^{{t}_{e}}$ (the average distance from the barycenter to the boundary of the core) and ${\overline{r} }_{m}^{{t}_{e}}$ (the average distance from the barycenter to the network boundary).

Then, we investigate the static properties of each metro in 2019. Network sizes range from 80 to 339, and they are roughly proportional to ${M}_{l}^{{t}_{e}}$ in the table. The degree has an average in the range [1.98, 2.31]$.$ Due to the physical constraints and the high cost of constructing a new metro line, ${\langle k\rangle }^{{t}_{e}}$ for metro networks are relatively small, similar ranges [2.18, 3.75] and [2.06, 2.22] can be found in Ref.^26^ and Ref.^12^, respectively, which reveals that the number of nodes and the number of edges is in the same order. The mean interstation distance is on average ${\overline{l} }^{{t}_{e}}\approx 1.6 \mathrm{km}$ in Table 1, which is larger than the mature metro networks globally (here, the metro networks in New York, Paris, London, and Chicago are considered the mature networks for those networks nearly unchanged in the last 20 years. The average ${\overline{l} }^{{t}_{e}}$ of them is less than 1.0 km). The total route length ${L}_{T}^{{t}_{e}}$ is an important indicator to represent the scale of the metro network. At the end of 2018, the Shanghai metro and Beijing metro became the most comprehensive system (641.39 km) and the second-longest metro system (619.10 km). Shenzhen and Guangzhou metro also joined the top 10 of the most extended metro systems. Five metro systems also exceed 200 km: Chengdu, Chongqing, Nanjing, Tianjin, and Wuhan.

The ${N}_{c}^{{t}_{e}}$ and ${\overline{r} }_{c}^{{t}_{e}}$, which give us the initial insight into the topological properties of the core, which are usually proportional to ${N}^{{t}_{e}}$. Besides that, ${\overline{r} }_{m}^{{t}_{e}}$ is about twice as large as the ${\overline{r} }_{c}^{{t}_{e}}$. Chongqing metro is an exception and has a relatively large ${N}_{c}^{{t}_{e}}$, the largest ${\overline{r} }_{c}^{{t}_{e}}$ and ${\overline{r} }_{m}^{{t}_{e}}$ because of the particular geography, Chongqing metro constructed long-distance connections for the dense population separated by mountainous and multiple river valleys as a famous inland mountain city. The descriptions of network indicators give a rough impression of the metro network structure in 2019. However, more information is needed to obtain a precise relationship with evolution. In the spirit of exploring the evolution of topological and efficient attributes, we try to understand how the topology of metro networks scales with time. As a result, we can explain the efficiency of metro networks from a historical perspective in the following subsections.

### Evolution of network properties

The network size characterizes the most straightforward measure of metro networks. To study the evolution of ${\varvec{N}}$, we show this quantity in Fig. 2. The rapid growth of nodes occurs at the earlier stage except for the older metro networks (i.e., Beijing metro and Tianjin metro). All the networks have been increasing sharply since 2010 in Fig. 2a. Besides, to further investigate the evolution of nodes across all the networks, it is worth looking at the inset in Fig. 2b. The sum of all stations (${{\varvec{N}}}_{{\varvec{s}}}$) for all cities increased by less than 550 from 1969 to 2009. The growth of metro scales generally reflects the development of the social-economic environment. The local economic power and the changing domestic political economy are significant motivations to develop metro systems in mainland China^27^. To counter slowing economic growth, the Chinese government has returned to the policy playbook that worked well after the 2008 recession: spending massive money on large infrastructure projects, including urban metro systems. Therefore, the past decade has witnessed a steeply increasing trend of stations with growing variations during 2010–2019 and the rapid growth of GDP shown in insets of Fig. 2a and b, respectively. This trend seems to be confirmed in the next five years after the National Development and Reform Commission had approved developing metro systems in mainland China at the start of 2018. It indicates convincingly that metro networks will become more significant in the future.

The historical development of metro networks investigated here is very different from one city to another leading to dispersion in the statistics of several stations $N$ given a particular year $t$ reflected by the ${N}_{\sigma }$. As noted above, the network extension depends on many parameters such as the local economy, inter-city competition, and domestic political economy that are different from one city to another. City networks may experience different development patterns based on their complex and comprehensive considerations. Therefore, to make growth comparable across all metro networks and different periods, we study time evolution for each city in terms of $N$.

**Figure 3:**
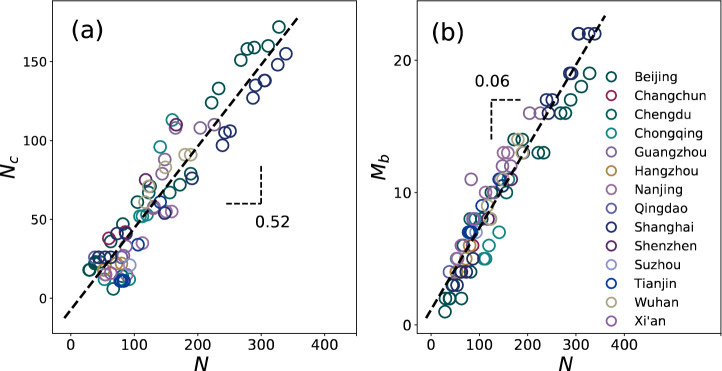
(**a**) Number of nodes in the core ($${N}_{c})$$ versus network size $$(N)$$. A linear fit (dashed line) gives $${N}_{c}=0.52N-7.41 ({R}^{2}=0.90)$$, or in other words, a minimum core (e.g., a triangle form) comprises at least 20 nodes. (**b**) The number of branches ($${M}_{b}$$) versus $$N$$ for metro networks in this article. $${M}_{b}\sim 0.06N ({R}^{2}=0.94)$$. $${N}_{c}$$, $${M}_{b}$$ and $$N$$ are aggregated by various cities over the years.

Let us first focus on the several topological properties of the evolution of core-branches structure. A parameter $\beta$ is defined as ${N}_{b}/N$ where ${N}_{b}$ and $N$ represent the number of stations on the branches and the network size for a given year $t$. Roth et al. found that $\beta$ converges to a limiting value ${\beta }_{\infty }\approx 0.45$^9^. Naturally, we take here a simple approach and assume that ${N}_{c}\sim N$ plotted in Fig. 3a. The data agree well with a linear fit ${N}_{c}\sim 0.52N ({R}^{2}=0.90)$ closely consistent with the ${\beta }_{\infty }$.

The growth of metro networks experiences expansion and intensification to satisfy the coverage and convenience^12^. Generally speaking, the intensifying mode increases the nodes in the core. The expansion mode adds new nodes on the branches (i.e., extending the branches or constructing new branches out of the core). An intuitive idea would be that the number of branches (${M}_{b}$) is proportional to the number of core nodes. As a result of the previous argument, we also consider ${M}_{b}\sim N$. A linear fit agrees relatively well with the data (${R}^{2}=0.94$, dashed line) shown in Fig. 3b.

### Temporal evolution and spatial organization of efficiency

As a crucial element in the public transportation system, the metro system is significant to the evolution of a city. It reflects the interplay of demography, economic development, and traffic demand over time. Generally speaking, a metro system improves efficiency considering local geological features and residents' commuting patterns. Characterizing the evolution of efficiency of the metro network is thus of the utmost importance in understanding the dynamic process taking place on them for further improving the efficiency and capacity of metro networks. The efficiency of a network can consider the average efficiency (${{\varvec{E}}}_{{\varvec{g}}}$ calculated in “Methods”) as an indicator of the traffic connection. It helps to grasp the topological properties of the metro networks on a global scope.

**Figure 4:**
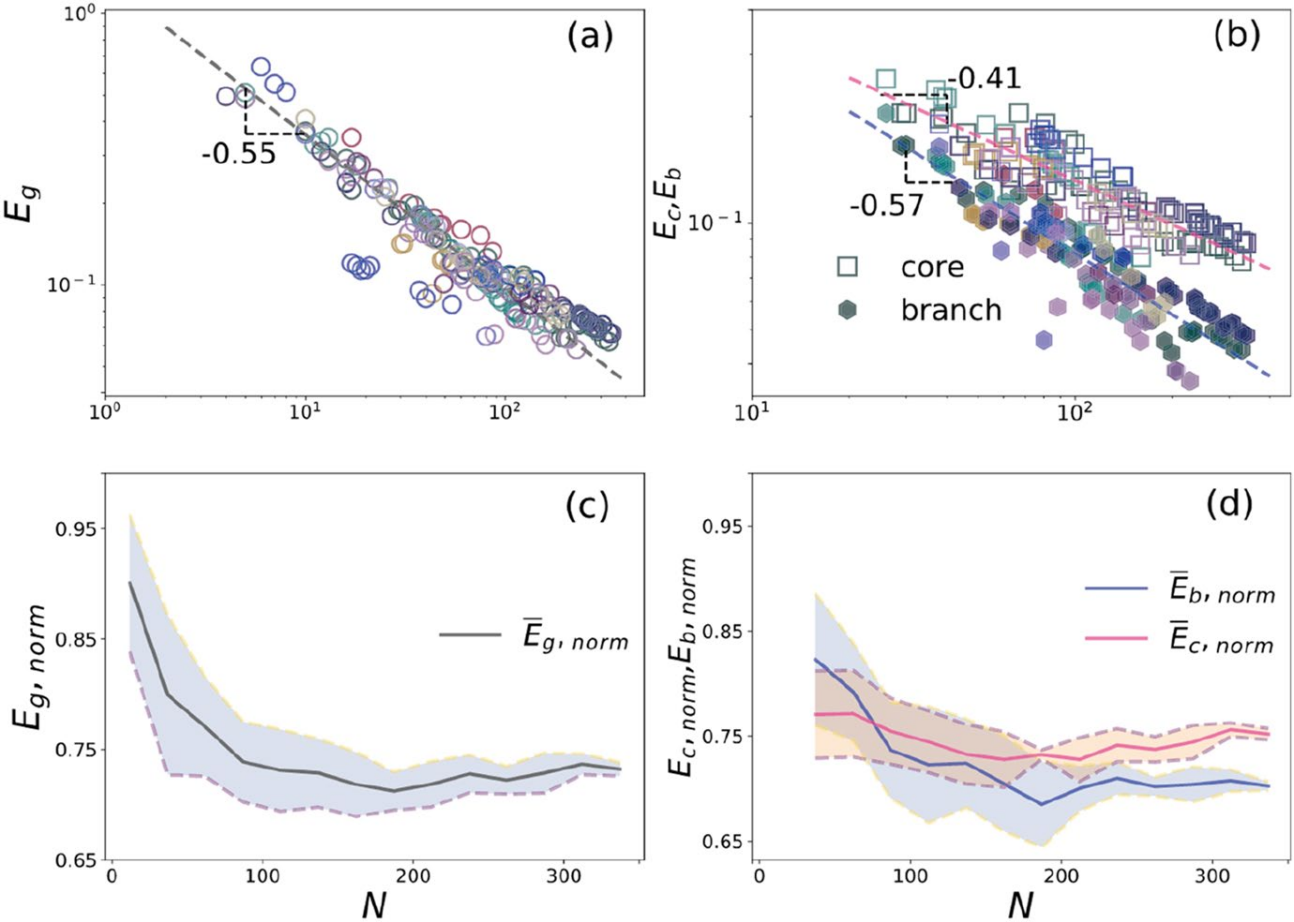
(**a**) Relation between global efficiency (${E}_{g}$) and network size ($N$). The grey dashed line shows the best power-law fit on the data points gives ${E}_{g}\sim {N}^{-0.55}$ (${R}^{2}=0.93$). (**b**) The unfilled circles show the average global efficiency of core nodes (${E}_{c}$) versus $N$. The filled hexagons are the average efficiency of the branch stations (${E}_{b}$) as a function of $N$ in metro networks. The two dashed lines in pink and blue represent the least-square fit assuming a power-law dependency, which gives respectively ${E}_{c}\sim {N}^{-0.41}$ (${R}^{2}=0.81$) and ${E}_{b}\sim {N}^{-0.57}$ (${R}^{2}=0.88$). (**c**) The evolution of ${E}_{g,norm}$, which characterizes the rescaling value of efficiency (averaged over 20 bins), and (**d**) same as (**c**) averaged over 20 bins and are showing the relationship between $N$ and ${E}_{c,norm}$, and ${E}_{b,norm}$ with the number of stations.

We find the dependence of ${E}_{g}$ on $N$ decreases with the expansion of networks, shown in Fig. 4a. A power-law fit function can charitably describe this relationship ${E}_{g}\sim {N}^{-0.55}$, which, interestingly, appears to be relatively independent of the peculiar historical, historical, and social-economical mechanisms associated with the growth of the city. We also verify the relationship in Chinese metro networks^22^, Barcelona and Madrid underground systems^23^, and 33 real-world metro networks^24^ estimated and compared by the network size, consistent with our findings. As we know, a metro system changes its network structure to improve transport efficiency and accessibility. Nevertheless, if we only focus on topological properties without considering the service coverage and passengers, we find that the spatial constraints deeply affect the evolution of efficiency, which decreases with the network sizes.

The evolution of metro networks and urban expansion is to satisfy the spatial distribution of traffic demands and economic activities. The underlying relationship between population distribution and economic development is one of the critical determinants of the formation and evolution of metro networks. For the past few decades, with the increase in congestion and land price, the employees and firms have moved out to the suburbs, which caused suburbanization and urban expansion to have been observed in large metropolitan areas all over the world^28^. Improvement of the transportation networks, especially metro networks, promote this phenomenon by offering rapid conveying commuters from suburban areas to urban areas and enabling the connection of entire urban areas. The common core and branches spatial pattern of metro networks responds to traffic demand generated by population development and economic development. Public transportation supports the development and growth of densely-populated metropolitan areas by facilitating labor movement outside or within the metropolitan area with better accessibility^29^. Taking this perspective, the development of metro networks aims to strengthen the efficiency of the core and make the nodes on the branches access the core.

Hence, we analytically calculate the average efficiency in the core (${E}_{c}$) and the average accessibility of the branches (${E}_{b}$) shown in Fig. 4b. When power-law regression analysis is carried out by the data of ${E}_{c}$, the exponent has a value of 0.41. The goodness of fit (${R}^{2}$) is calculated to be 0.81. By adapting the data for ${E}_{b}$, the exponent becomes 0.57, with the goodness of fit becoming 0.88. As expected, the ${E}_{c}$ decreases slower than the ${E}_{b}$ and ${E}_{g}$, which indicates that the expansion of metro networks intentionally enhances the average accessibility in the core. The relative disparity between ${E}_{c}$ and ${E}_{b}$ is enlarging. In other words, the evolution of the network structure involves an increase in the relative ratio of ${E}_{c}$. These results are consistent with the previous arguments and test the relationships between ${E}_{c}$ and ${E}_{b}$, sharing a typical relationship between the evolution of metro structure and the urbanization process.

According to the Eq. (2) the value of ${E}_{g}$ depends on the pairs of shortest path lengths in the metro network. As a typical spatial network, the lengths of edges are embedded in space, which may be affected by various geographical and historical activities^30^. Hence, to observe the normalization of global efficiency, we try to generalize the efficiency to capture and compare the transportation efficiency of metro systems. To this end, we rescale the value of global efficiency in $\left[\mathrm{0,1}\right]$ by the ideal proxy ${G}_{\mathrm{ideal}}$ [calculated by Eq. (3)] for the normalization of the ${E}_{g}$ (i.e., ${E}_{g,norm}$)^31^. The average value of ${E}_{g,norm}$ (${\overline{E} }_{g,norm}$) and its dispersion as well plot in Fig. 4c. ${\overline{E} }_{g,norm}$ seem to decrease with $N$ and stabilize slowly to a narrow range of value in $\left[0.71, 0.75\right]$. Both the ${\overline{E} }_{c,norm}$ and the ${\overline{E} }_{b,norm}$ display moderate variations towards a region of stable values when $N\ge 200$. It is worth to be mentioned that the ${\overline{E} }_{b,norm}$ is always smaller than ${\overline{E} }_{c,norm}$ when $N\ge 80$. We contribute the phenomenon to a densification process connecting the stations and increasing their efficiency with the increasing stations in the core area.

The metro network is not a closed system, as it can be considered only part of the multilayer network in a city. The local efficiency of the metro network will be vastly increased when extending the transportation system. As shown in Boston^26,32^, the additional subnetworks like the bus and tram networks significantly impact the overall network properties. Here, we only provide quantitative information on the efficiency characteristics of evolving metro systems, whose structures sprawl in two-dimensional space. Because of the space restrictions and relatively high construction cost, the global efficiency cannot be improved for lacking high redundancy in connecting two metro stations. However, with urban growth, metro networks are experiencing both expansions in the branches and intensification in the core area to satisfy the traveling demand of residents gradually. Our measurements find common characteristics of evolving metro networks and explain the underlying construction principles of transportation systems from the topological perspective.

**Figure 5:**
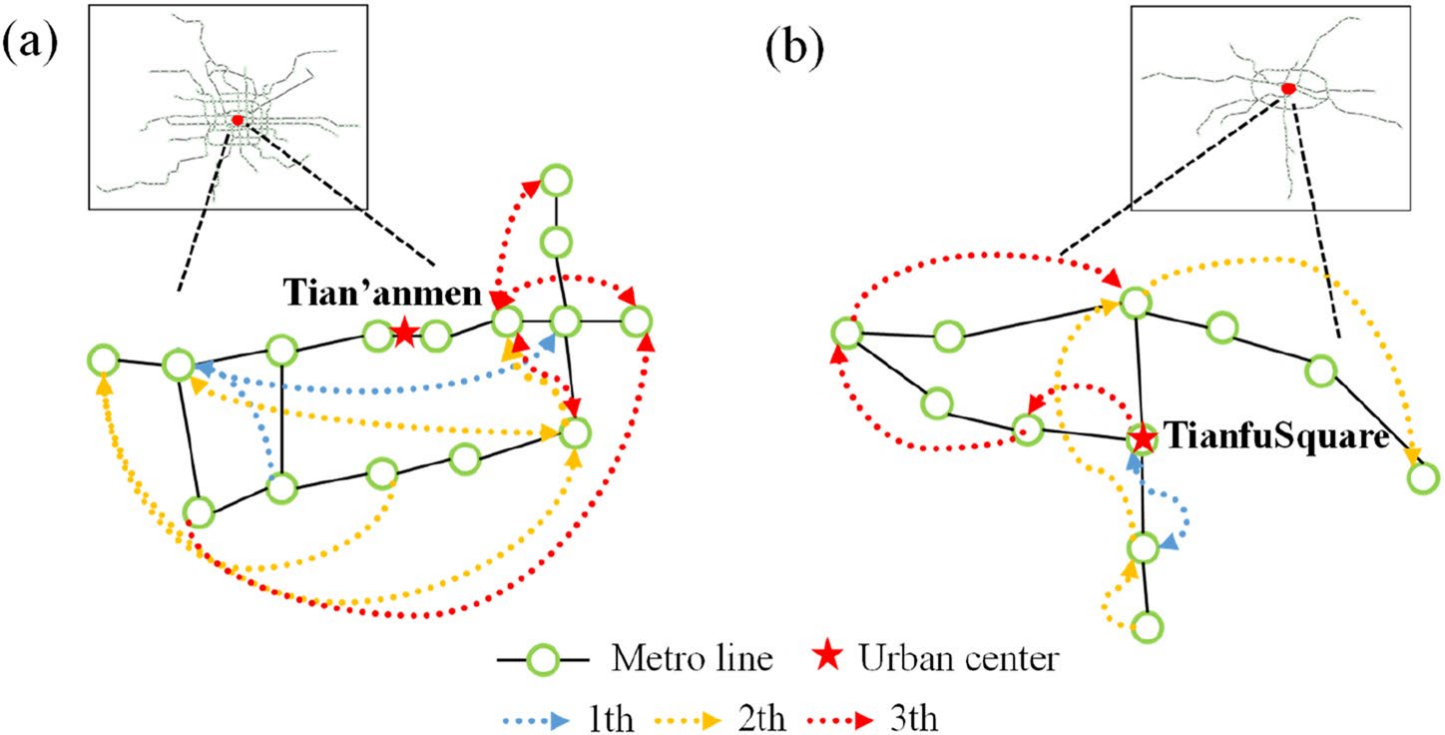
The time-varying top 3 efficient stations in Beijing metro (**a**) and Chengdu metro (**b**). Beijing and Chengdu’s urban centers (marked as redstarts) are Tian’anmen and Tianfu Square.

The results presented in the previous subsections prompt us to study the evolution of the highly efficient stations. The metro system keeps evolving under social and economic environments with time due to urban expansion. Therefore, one may expect that the most important stations of the metro network will also evolve accordingly. Taking Beijing metro and Chengdu metro as examples, we investigate the top 3 vital stations (Fig. 5a,b). One can conclude that the highest efficient stations are essentially different from the early stage to the current stage of the metro system navigated via the arrows in different colors. However, the top 3 stations in various stages continually surrounded the urban center (shown in the redstarts in Fig. 5). The area consisting of the most efficient nodes in history is in a small coverage area in the core, as shown in Fig. 5.

We further inspect the spatial distribution of metro networks' most efficient nodes. Following earlier studies on the fractal aspects of metro networks^19^ and spatial distribution of the highest influential nodes^5,33,34^, we analyze the nodal organization effect quantitatively by considering the top $p$ percent of highest efficient nodes less than or equal to radius $r$ ($r>0$) originated from centers defined in “Methods”.

**Figure 6:**
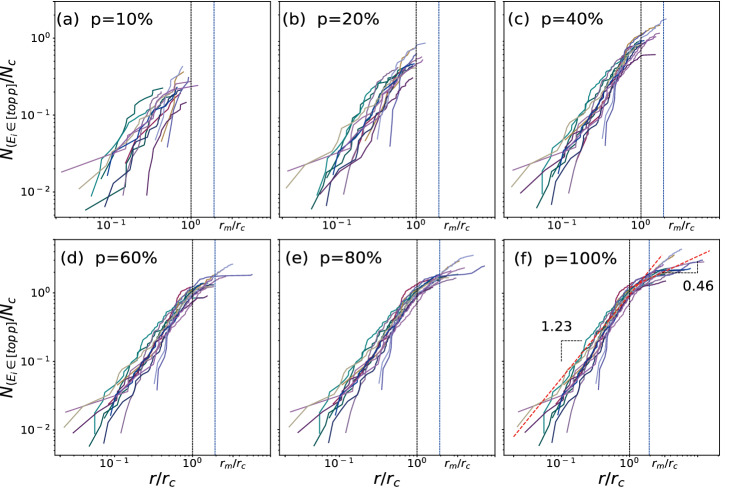
Highly efficient nodes in space. (**a**) For $t=2019$ (${t}_{e}$), the top 10% highest efficient nodes in a disk of radius $r$ as a function of the rescaled variable $r/{r}_{c}$. (**b**) Same as (**a**) but showing the top 20%. (**c**) and (**d**) represent the top 40% and 60%, respectively. (**e**), and (**f**) represent the top 80% and all nodes (The dashed lines serve here as a guide to the eye). The physical distances are originated from the center of the core defined in “Methods”.

We calculate the efficiency inside a disk of radius $r,$ as shown in Fig. 6. First, let us focus on subfigure (a) for the spatial distribution of the metro network's top 10% efficient nodes. Most of them dwell in the core for $r\le {\overline{r} }_{c}^{{t}_{e}}$, which enhances the conclusions in Fig. 5. When $p$ increases to 20%, barely find nodes for $r>{\overline{r} }_{c}^{{t}_{e}}$, the most influential nodes intensify in the most massive core range. Moreover, the distribution of the highest efficient nodes tends to have a similar shape. However, for $40\mathrm{\%}\le p\le 80\%$, most nodes in the core are included (shown in Table 1 and Fig. 4), the distribution of ${N}_{{E}_{i}\in [\mathrm{top} p]}$ converges to a similar shape corresponding to a uniform core in Fig. 6c–e. It is must be noted that some medium-sized cities (e.g., Suzhou metro, Qingdao, and Hangzhou) have a relatively small core occupying less than an average 30% number of nodes (shown in Table 1). When $p$ is more significant than 60%, the top $p$ efficient nodes will include forks on the branches, and nodes near the core appear at the distance $r>{\overline{r} }_{m}^{{t}_{e}}$ in these networks.

Spatial distributions for the top 100% accessible nodes are, actually, the spatial measures of the number of stations at a given distance $r$. In Fig. 6f, the spatial distance regime $r<{\overline{r} }_{c}^{{t}_{e}}$ is well described by the distance $r$, the red dashed line (a guide to the eye) represents a power law $N(r)\sim {r}^{1.23}$ (${R}^{2}=0.93$). The next regime $r>{\overline{r} }_{c}^{{t}_{e}}$ displays different behaviors with $r$ ($N(r)\sim {r}^{0.46}$, ${R}^{2}=0.71$) controlled by the interstation spacing on branches. These topological and spatial features can also find in the early study^9^. However, there is a slight difference in the definition of the barycenter. They excluded the Chicago metro for the applying restriction of barycenter due to the presence of a lake. It is worth mentioning that some metro networks investigated here, such as Shenzhen and Qingdao, have similar geographical limitations. However, they also appear with scaling property for considering the center as the geographical barycenter in the core (shown in Fig. 6f). Our results show that the barycenter of the core has its advantage in depicting the spatial organization of metro stations. The nodes with the highest efficiency reside within the core of metro networks and gradually expand outward nodes on the branches.

## Conclusions

The main objective is to study the evolution of efficiency and the structural features of complex metro systems. The most extensive metro networks exhibit a similar temporal decrease efficiency around increasing stable values for the core in the long term; the spatial organization of efficient nodes converges to a similar structure. Such work can serve as a basis for assessing the properties of metro networks. A better understanding of the common mechanisms allows simple transportation modeling and planning. It is essential to account for these underlying weighted architectures to gain fundamental insights into the hidden construction principles and mechanisms used to transform, process, and exchange information in transportation systems^35^.

Urbanization promotes suburbanization and urban expansion, which brings a rapid traffic demand for connecting urban and suburban areas. The typical structure of metro networks consists of a core and branches formed by satisfying the rapidly increasing traffic demand. The nodes with the highest efficiency are located within the core and gradually expand outward on the branches from the center, reflecting the difference in economic role in different areas. The evolution of the metro network is closely related to urban expansion and therefore influenced by numerous factors such as geographical, historical, and social origin. We present evidence that the evolution of efficiency in various cities shares common statistical properties based on empirical observations. The topological properties of the metro systems and the existence of such relations suggest that underlying, universal mechanisms are at play during their evolution in the development of cities.

However, our analyses have some limitations. First, we consider only the evolution of network indicators that may affect the connection and efficiency. The central planning and economic-social factors are just mentioned, not investigated for quantifying the effect. Other interesting data related to the topology, such as traffic flow, population distribution, and land use, which show a strong relationship with metro networks^36^, may enrich our future study. For instance, the influence of the subway system on the population was revealed in a recent study by employing ridership and efficiency as indicators and found that only commercial activities are substantially coupled to the subway system, which sheds light on the interplay of the metro network and land use^37^. Second, as a crucial component of the public transportation systems, the metro network is one layer of the multimode network and co-evolving with other transportation modes. Therefore, adding the time evolution of the bus and road networks in our analyses will acquire a better and deep understanding of the evolution of metro networks. Finally, efficiency measures the capacity of mobility in terms of people, freight, or information, while we only measure geographic efficiency. Developing the concept of distance weighted by traffic flow or train frequency will provide additional implications for the evolution of metro networks in further extensions.

Our understanding of how metro networks evolve is still inadequate despite decades of effort. The results of our study provide, for the first time, empirical evidence on the evolution of efficiency in metro networks, which is helpful for understanding and modeling the evolution of metro networks. The spatial expansion and efficiency evolution of metro networks always accompany urban growth, and the urban extension stimulates changes in traffic demand, land use, and population distribution, reshaping the spatial structure of urban transportation networks. We contribute our study presented here to represent a crucial step towards a better understanding of these multiple relationships, particularly in the future realization of the smart city. We hope our findings contribute to economic efforts, urban planning, and policy-making in cities' current rapid advancement contexts.

## Methods

### Metro network data

The first step toward analyzing the evolution of metro systems is to collect data on their structures. We gathered and analyzed data related to the metro networks from 1969 to 2019. The primary topological data were obtained from Wikipedia (http://www.wikipedis.org). For each metro network, a specific page per metro line includes various information such as the station names, the first date of operation for each station, the distance between neighbor stations, and the total length of each line. This information allows for a complete reconstruction of the metro lines and corresponding operating stations at any time.

For a given year $t$, the network building process is as follows. The list of operating stations and lines at year $t$ was first established. Stations are connected between contiguous stations and metro lines, and the rail distance of neighbor stations will be added to the graph. Then, those independent line topologies are gathered into a weighted and undirected graph. As a transfer station may have different names in the dataset, metro maps for 2019 re-index each station. Simultaneously, to avoid any other conflicts, we join stations spatially using latitude and longitude extracted from the Baidu map (https://map.baidu.com/) to double-check the structure. It is worth mentioning that the metro maps in 2019 cannot process all the stations for each year of their existence. For example, a station that closed before 2019 and remained closed (e.g., Xinhua Road station in Tianjin metro) will be handled separately. Eventually, the topological structure of metro networks is modeled in L-space as an undirected and weighted graph $G$ with $N$ vertices (stations) and $E$ edges (rail tracks) corresponding to year $t$.

### Definition of core-branch structure and geographical barycenter

Metro network in the early stage always begins with linear, circular, or star form, then becomes increasingly large and complex. Although it is challenging to classify the form of the metro network into a single topology category, it is evident that its form has changed from a simple to a more complicated form. In 2012, Roth et al.^9^ found that the topological structures of large metro networks in the world gradually developed into the shape with a denser core (i.e., surrounded by a circular line such as a ring line or coalescing a series of lines) and branches reaching out to the suburbs or areas further from the core.

Metro networks investigated here display a core-branches structure through spatial representations plotted by the relative location of stations in Fig. 1. The core in the metro network (light green nodes in Fig. 1) is a $k$-core subgraph with $k=2$ obtained by *k*-core decomposition^25^, where $k$ is the nodal degree. Precisely, iteratively remove all nodes with degree $k=1$ in $G$. The remaining graph is the *k*-core subgraph considered the metro network's core, which seems to be located in the center of the spatial topology in Fig. 1. All the removed nodes lay at the periphery and branches of the metro network. This measuring method of core-branches structure is also applied in Ref.^9^.

The geographical barycenter is always considered as the center defined as the average location of all the stations. The geographical barycenter (marked in black nodes in Fig. 1) is close to the center nodes (e.g., spatial centrality and betweenness centrality) used as the starting point to determine the spatial distance^5,30^. However, this definition of barycenter has several restrictions on the application of the metro network: First, the metro network is always considered a planar spatial graph formed with an anisotropic shape for the most extensive networks (e.g., Beijing metro and Shanghai metro). While several metro networks may not have this characteristic due to geographical constraints such as the sea in the south of Shenzhen, this may cause the movement of barycenter with the evolution of the network and generate deviations for measuring the spatial organization of the network^9^. Besides the developing metro networks (e.g., Qingdao metro and Suzhou metro), the barycenter is not surrounded by the core nodes, as shown in Fig. 1. The applying restriction of barycenter is attributed to the shape of branches is not isotropic homogeneity. Here, we redefine the barycenter as the average location of all the stations in the core. If there is no core for a metro network, the barycenter is back to the original definition. Herein, the geographical barycenter refers to the average location of the core stations, that is, the barycenter in the core (red nodes in Fig. 1).

### The efficiency of the metro network

The network efficiency is an indicator of exchange information based on the shortest path calculated as path distances or the opportunity costs at a station to other stations in a metro network. The average efficiency of node ${\varvec{i}}$ (${{\varvec{E}}}_{{\varvec{i}}}^{{\varvec{t}}}$) for a given year ${\varvec{t}}$ is calculated as:

1 $${E}_{i}^{t}=\frac{1}{N-1}\sum_{j,j\ne i}\frac{1}{{d}_{ij}},$$

where ${d}_{ij}$ is the shortest path distance between node $i$ and node $j$. The connectivity efficiency between node $i$ and node $j$ is measured as the inverse of the shortest path length $(1/{d}_{ij})$. This measure can be determined even if there is no path between two nodes, as in the case of a disconnected graph: ${\mathrm{log}}_{{d}_{\mathit{ij}}\to \infty }1/{d}_{ij}=0$. ${E}_{i}$ is normalized to all the other nodes ($N-1$) for comparison. The higher ${E}_{i}$ indicates higher efficiency, i.e., the shorter distance is covered to reach potential destinations from node $i$.

The global efficiency ${E}_{g}^{t}$ is normalized to the network size ($N$) corresponding to year $t$, an index of the effectiveness of information exchange over the network to evaluate topological transportation efficiency^32^.

The value of ${E}_{g}$ depends on the scale of path distance and, in general, ${E}_{g}\epsilon [0,\infty ]$, which cannot be effortlessly generalized to weighted networks. For this reason, Latora et al.^38^ rescaled the value of global efficiency in $\left[0, 1\right]$ by considering an idealized proxy of $G$, ${G}_{ideal}$, having maximum efficiency. ${E}_{g,ideal}$ is based on pairwise physical distances ${l}_{ij}$:

2 $${E}_{g,ideal}^{t}=\frac{1}{N\left(N-1\right)}\sum_{j\ne i}\frac{1}{{l}_{ij}}.$$

For all $i, j \epsilon V$, constraint ${l}_{i,j}\le {d}_{i,j}$ is always satisfied whether $G$ is disconnected or connected and, hence, ${E}_{g,ideal}\ge {E}_{g}$. A correct normalization of ${E}_{g}$ is then possible to use ${E}_{g,ideal}$ resulting from a physically-grounded enrichment procedure independent from the scale of form. The normalized global efficiency can be then calculated as:

3 $${E}_{g,norm}^{t}=\frac{{E}_{g}^{t}}{{E}_{g,ideal}^{t}}.$$

For metro networks, the physical distances are well-defined by the underlying geometry and computed as corresponding great-circle distance about spatial coordinates of stations^36^.

## Data Availability

The data and code supporting the findings of this study are available from the corresponding authors upon reasonable request.
